# Exploring the antimicrobial resistance profiles of WHO critical priority list bacterial strains

**DOI:** 10.1186/s12866-019-1687-0

**Published:** 2019-12-23

**Authors:** Benjamin Havenga, Thando Ndlovu, Tanya Clements, Brandon Reyneke, Monique Waso, Wesaal Khan

**Affiliations:** Department of Microbiology, Faculty of Science, Stellenbosch University, Private Bag X1, Stellenbosch, 7602 South Africa

**Keywords:** *Acinetobacter baumannii*, *Pseudomonas aeruginosa*, *Escherichia coli*, *Klebsiella pneumoniae*, Colistin resistance, Surfactin

## Abstract

**Background:**

The antimicrobial resistance of clinical, environmental and control strains of the WHO “Priority 1: Critical group” organisms, *Acinetobacter baumannii*, *Escherichia coli*, *Klebsiella pneumoniae* and *Pseudomonas aeruginosa* to various classes of antibiotics, colistin and surfactin (biosurfactant) was determined.

**Methods:**

*Acinetobacter baumannii* was isolated from environmental samples and antibiotic resistance profiling was performed to classify the test organisms [*A. baumannii* (*n* = 6), *P. aeruginosa* (*n* = 5), *E. coli* (*n* = 7) and *K. pneumoniae* (*n* = 7)] as multidrug resistant (MDR) or extreme drug resistant (XDR). All the bacterial isolates (*n* = 25) were screened for colistin resistance and the mobilised colistin resistance (*mcr*) genes. Biosurfactants produced by *Bacillus amyloliquefaciens* ST34 were solvent extracted and characterised using ultra-performance liquid chromatography (UPLC) coupled to electrospray ionisation mass spectrometry (ESI–MS). The susceptibility of strains, exhibiting antibiotic and colistin resistance, to the crude surfactin extract (cell-free supernatant) was then determined.

**Results:**

Antibiotic resistance profiling classified four *A. baumannii* (67%)*,* one *K. pneumoniae* (15%) and one *P. aeruginosa* (20%) isolate as XDR, with one *E. coli* (15%) and three *K. pneumoniae* (43%) strains classified as MDR. Many of the isolates [*A. baumannii* (25%), *E. coli* (80%), *K. pneumoniae* (100%) and *P. aeruginosa* (100%)] exhibited colistin resistance [minimum inhibitory concentrations (MICs) ≥ 4 mg/L]; however, only one *E. coli* strain isolated from a clinical environment harboured the *mcr-1* gene. UPLC-MS analysis then indicated that the *B. amyloliquefaciens* ST34 produced C_13–16_ surfactin analogues, which were identified as Srf1 to Srf5. The crude surfactin extract (10.00 mg/mL) retained antimicrobial activity (100%) against the MDR, XDR and colistin resistant *A. baumannii*, *P. aeruginosa*, *E. coli* and *K. pneumoniae* strains.

**Conclusion:**

Clinical, environmental and control strains of *A. baumannii*, *P. aeruginosa*, *E. coli* and *K. pneumoniae* exhibiting MDR and XDR profiles and colistin resistance, were susceptible to surfactin analogues, confirming that this lipopeptide shows promise for application in clinical settings.

## Background

Bacterial antibiotic resistance is a global public health crisis and in 2017, the World Health Organisation (WHO) created a list of 12 genera and/or families, which were prioritised for the development of alternative antimicrobials into the categories critical, high and medium. Carbapenem-resistant *Acinetobacter baumannii* (*A. baumannii*) was classified as one of the main “Priority 1: Critical group” organisms [1] as it is the primary opportunistic pathogen implicated in nosocomial infections such as; bacteraemia, pneumonia, endocarditis, meningitis and catheter associated urinary tract infections (UTIs) [2–4]. It is also the primary species detected and isolated from hospital environments, including intensive care units (ICUs), with many other *Acinetobacter* species frequently isolated from soil, water, vegetables and animal sources [5]. *Pseudomonas aeruginosa* (*P. aeruginosa*), the second carbapenem-resistant bacterium listed in the “Priority 1: Critical group”, can be found in a diverse range of habitats and is classified as an opportunistic pathogen in animal or plant hosts [6]. Several *P. aeruginosa* strains have also been isolated from hospital environments and have been implicated in nosocomial infections. This opportunistic pathogen primarily infects the immunocompromised and elderly and has been identified as the causative agent in burn wound infections, as the biofilm coloniser of medical devices and causes lung infection in cystic fibrosis patients [7, 8]. A study by Tam et al. [9] also revealed that patients infected with MDR *P. aeruginosa* were hospitalised for a significantly longer time period and were at an increased risk for 30-day mortality.

*Escherichia coli* and *K. pneumoniae* are members of the family Enterobacteriaceae, which is classified as carbapenem-resistant, 3rd generation cephalosporin-resistant bacteria [1]. *Escherichia coli* is predominantly found in soil and aquatic environments; however, it is also present in the lower gastrointestinal tract of warm-blooded animals and in reptiles [10, 11]. Enteric or intestinal *E. coli* strains can be classified into pathotypes based on phenotypic traits and specific virulence factors [12], with UTI’s, gastroenteritis, meningitis, peritonitis and septicaemia in hospital settings commonly caused by various strains of *E. coli* [13, 14]. *Klebsiella pneumoniae* is an opportunistic pathogen found in several environmental niches such as soil, plants and water. It is a common skin commensal and inhabits the gastrointestinal tract and nasopharynx of animals and humans [15]. Due to its presence on or in humans and animals it is responsible for several systemic infections such as UTIs, bacteraemia and pneumonia [16].

The global emergence of clinical infections due to multidrug resistant (MDR) and extreme drug resistant (XDR) strains of the “Priority 1: Critical group” has subsequently led to a resurgence of the last-resort antibiotic colistin (polymyxin E) [17–19]. However, while colistin resistance was previously considered to be a rare chromosomal mutation, in 2015 a novel method of plasmid mediated colistin resistance was documented, when a mobilised colistin resistance gene (*mcr-1*), identified on an IncI2 plasmid, pHNSHP45, was isolated by Liu et al. [20] from pigs in China. A study performed by Newton-Foot et al. [21] detected colistin resistance encoded by the *mcr-1* gene in isolates of *E. coli* and *K. pneumoniae*, amongst others, obtained from eight different hospitals in the Western Cape region of South Africa. Following the detection of the *mcr-1* gene, several other *mcr* genes have been detected: *mcr-2* harbouring *E. coli* isolated in Belgium [22]; *mcr-3* harbouring *E. coli* isolated from swine in China [23]; *mcr-4* harbouring Enterobacteriaceae species (*E. coli* and *Salmonella enterica* serovar Typhimurium) isolated form pigs in Belgium, Italy and Spain [24]; and *mcr-5* carrying *Salmonella Paratyphi* B dTa + isolated from animals in Germany [25]. While, the mechanism of colistin resistance in *A. baumannii* and *P. aeruginosa* has not been fully elucidated, the rate at which bacterial pathogens are exhibiting resistance to this last-resort treatment is rapidly increasing, which implies that novel strategies or alternative antimicrobials are urgently required to combat MDR and XDR bacteria.

Biosurfactants are secondary metabolites, synthesised non-ribosomally by bacteria, yeast and fungi during the exponential and/or stationary growth phases [26, 27]. They can be classified into six major groups, however glycolipids (e.g. rhamnolipids, sophrose lipids, trehalose lipids) and lipopeptides (e.g. surfactin, viscosin, serrawettin) are of extreme interest to the medical field as various classes exhibit broad-spectrum antimicrobial activity [28]. The widely studied lipopeptide, surfactin, has subsequently been utilised as an anti-inflammatory, anti-adhesive, antiviral and antibacterial agent and has also been employed in bioremediation strategies [27, 29]. This lipopeptide is generally synthesised by *Bacillus* species and consists of an amphipathic cyclic heptapeptide with a chiral sequence (L-Glu, L-Leu, D-Leu, L-Val, L-Asp, D-Leu, L-Leu) linked to a β-hydroxy fatty acid group comprised of 13 to 16 carbon atoms [30, 31]. Ndlovu et al. [27] investigated the antimicrobial activity of a crude surfactin extract obtained from a *Bacillus amyloliquefaciens (B. amyloliquefaciens*) ST34 strain against various pathogenic bacteria, fungi and yeast. The extract produced pronounced antimicrobial activity against the antibiotic resistant *Staphylococcus aureus* (Methicillin Resistant *S. aureus* – MRSA), *E. coli* and the opportunistic pathogenic yeast strain *Candida albicans.*

The primary aim of the current study was to explore the antimicrobial resistance profiles of clinical, environmental and control strains of the WHO “Priority 1: Critical group” organisms, *A. baumannii*, *P. aeruginosa*, *E. coli* and *K. pneumoniae*. Various experimental tasks were performed to achieve this aim including the isolation of *A. baumannii* from environmental samples. The antibiotic resistance profiles of the *A. baumannii*, *P. aeruginosa*, *E. coli* and *K. pneumoniae* strains were subsequently analysed against an array of antibiotics, whereafter the colistin resistance profile of the isolates exhibiting multidrug resistance was determined. Finally, the susceptibility of the clinical, environmental and control strains, exhibiting multidrug and colistin resistance, to the crude extract of surfactin was determined.

## Results

### Isolation of *A. baumannii* and sequence-based identification of test strains

Based on morphological characterisation, three isolates from the Stellenbosch WWTP, seven isolates from the Plankenburg River and six isolates from the stream in Enkanini informal settlement were presumptively identified as *A. baumannii*. In order to confirm the identity of these isolates, as well as the reference and clinical strains of *A. baumannii* and the reference, clinical and environmental strains of *E. coli*, *K. pneumoniae* and *P. aeruginosa* (obtained from the various culture collections), genus or species-specific conventional PCR and DNA sequencing was performed. It should be noted that the strains used in the current study were assigned code name identifiers as outlined in Additional file 1: Table S1. Henceforth the isolates will be referred to by their code identifiers. Furthermore, the GenBank accession numbers are listed in Additional file 1: Table S1.

Conventional PCR and sequencing of the *A.b_hyp* gene (545 bp) identified one of the 16 presumptive isolates as *A. baumanii* (CP030106.1). The *A. baumannii* isolate (AB 6) was obtained from the Enkanini informal settlement stream sample using growth media specific for *Campylobacter* species [32]. Sequencing analysis then confirmed the identity of the *A. baumannii* ATCC reference strain (AB 1) and the clinical isolates (AB 2 to AB 5; Additional file 1: Table S1).

Sequencing of the amplified *phoA* gene (903 bp) confirmed the identity of all the *E. coli* reference (EC 1), clinical (EC 2 and EC 3) and environmental (EC 4 – EC 7) isolates used in the current study (Additional file 1: Table S1). Similarly, the identity of all the *K. pneumoniae* isolates [reference (KP 1), clinical (KP 1 – KP 3) and environmental (KP 5 – KP 7)] was confirmed by conventional PCR and sequencing of the *Klebsiella gyrA* gene (383 bp), while the identity of all the *P. aeruginosa* isolates [reference (PA 1), clinical (PA 2 and PA 3) and environmental (PA 4 and PA 5)] used in the current study was confirmed using conventional PCR and sequencing targeting the16S rRNA region (618 bp) within the *Pseudomonas* genome.

As a quality control step, conventional PCR and sequencing thus confirmed the identity of the reference, clinical and environmental isolates as *A. baumannii* (*n* = 6), *E. coli* (*n* = 7), *K. pneumoniae* (*n* = 7) and *P. aeruginosa* (*n* = 5).

### Kirby-Bauer antibiotic assays

**Fig. 1:**
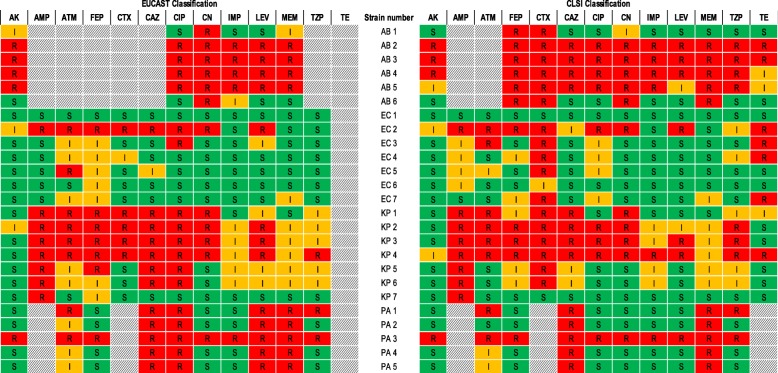
Antibiotic susceptibility profiles of all test organisms as determined by disc diffusion assays and classified according to the EUCAST [33] and CLSI [34] guidelines. Resistant (R; Red); Intermediate (I; Orange); Susceptible (S; Green); Thin diagonal stripes = Data not available (N/A), antibiotics not tested. Amikacin (AK), Ampicillin (AMP), Aztreonam (ATM), Cefepime (FEP), Cefotaxime (CTX), Ceftazidime (CAZ), Ciprofloxacin (CIP), Gentamicin (CN), Imipenem (IMP), Levofloxacin (LEV), Meropenem (MEM), Piperacillin-tazobactam (TZP), Tetracycline (TE)

The respective bacterial isolates (*n* = 25) were subjected to various antibiotics (Additional file 1: Table S2) [33, 34], with the results of the Kirby-Bauer Antibiotic Assays summarised in Fig. 1 and Additional file 1: Figure S1. Zones of inhibition were measured and compared to the European Committee on Antimicrobial Susceptibility Testing (EUCAST) [33] and Clinical and Laboratory Standard Institute (CLSI) [34] breakpoints.

The antimicrobial susceptibility analysis indicated that the *A. baumannii* isolates AB 1 (reference strain) and AB 6 (environmental strain) exhibited low resistance towards the antibiotics analysed (Fig. 1). In contrast, based on the EUCAST [33] breakpoints, the clinical isolates AB 2 to AB 5 were resistant to 100% of the tested antibiotics (Fig. 1) and based on the CLSI [34] breakpoints, AB 2 to AB 4 (exception of tetracycline where an intermediate result was recorded) were resistant to all the antibiotics analysed; while AB 5 displayed resistance to 73% (8/11) of the analysed antibiotics. The four clinical *A. baumannii* isolates (AB 2 – AB 5) thus displayed resistance to β-lactam antibiotics, fluoroquinolones and aminoglycosides as well as to carbapenems and were classified as XDR.

According to both the EUCAST [33] and CLSI [34] breakpoints, the reference EC 1 isolate was susceptible to 100% of the tested antibiotics (Fig. 1). Similarly, isolates EC 3 to EC 5 exhibited intermediate susceptibility and susceptibility to most of the antibiotics analysed (Fig. 1) [33, 34]. In contrast, the clinical EC 2 isolate was resistant to 67% (8/12), intermediately susceptible to 8% (1/12) and susceptible to 25% (3/12) of the tested antibiotics according to the EUCAST [33] breakpoints. In accordance with the CLSI [34] breakpoints, EC 2 also displayed resistance to 62% (8/13) of the antibiotics analysed, while intermediate susceptibility and susceptibility was recorded against 23% (3/13) and 15% (2/13), respectively, of the tested antibiotics. The clinical *E. coli* isolate EC 2 thus exhibited resistance to β-lactams, fluoroquinolones and aminoglycosides and was characterised as MDR.

While the environmental KP 5 to KP 7 strains displayed susceptibility and intermediate susceptibility to most of the antibiotics analysed, the reference KP 1 isolate displayed resistance to 58% (7/12), intermediate susceptibility to 17% (2/12) and susceptibility to 25% (3/12) of the tested antibiotics according to the EUCAST [33] breakpoints (Fig. 1). However, based on the CLSI [34] breakpoints, KP 1 displayed resistance to 38% (5/13), intermediate susceptibility to 23% (3/13) and susceptibility to 38% (5/13) of the tested antibiotics. Based on the EUCAST [33] and CLSI [34] breakpoints, the clinical KP 2 and KP 3 strains displayed similar profiles and were resistant to the majority of the antibiotics tested (Fig. 1). Furthermore, the clinical KP 4 exhibited the same antibiotic profile as KP 3, however, it was also resistant to piperacillin-tazobactam (TZP) [33]. Based on the CLSI [34] breakpoints, isolates KP 3 and KP 4 again displayed similar antibiotic profiles, with the exception that KP 3 was susceptible to amikacin while intermediate susceptibility was recorded for KP 4, and KP3 was susceptible to tetracycline (TE), while KP 4 was resistant. The reference KP 1 and clinical KP 2 and KP 3 isolates were subsequently classified as MDR as they exhibited resistance to the three antibiotic classes β-lactams, fluoroquinolones and aminoglycosides, while KP 4 was classified as XDR and exhibited resistance to the three antibiotic classes including carbapenems [imipenem (IMP)].

According to the EUCAST [33] breakpoints all *P. aeruginosa* isolates (PA 1 – PA 5) exhibited resistance to the antibiotics ceftazidime (CAZ), ciprofloxacin (CIP), levofloxacin (LEV) and meropenem (MEM). However, based on the CLSI [34] breakpoints the *P. aeruginosa* isolates were only classified as resistant to ceftazidime (CAZ) and meropenem (MEM). Both the EUCAST [33] and CLSI [34] breakpoints classified the clinical isolate PA 3 as 100% resistant to all tested antibiotics and it was subsequently classified as XDR.

### Colistin resistance of isolates exhibiting antibiotic resistance

Based on the results obtained for the antibiotic susceptibility assays, the MIC of 16 isolates (reference and representative MDR and XDR strains) (Table 1), against the last resort antibiotic colistin, was determined. Based on the EUCAST [33] and CLSI [34] breakpoints, a colistin MIC of ≥4 mg/L was recorded for 11 of the 16 isolates analysed, while PA 2 displayed a colistin MIC of 8 mg/L (Table 1). The reference isolate AB 1, the clinical isolate AB 2, the environmental isolate AB 6 and the clinical isolate EC 2 exhibited colistin MICs of 2 mg/L based on the EUCAST [33] and CLSI [34] breakpoints. As varying degrees of colistin resistance were recorded for the test isolates, colistin resistance mediated by the *mcr*-genes was determined [19]. Subsequently, all the organisms were screened for the plasmid-mediated mobilised colistin resistance genes (*mcr-1* to *mcr-4*) using conventional PCR. Results indicated that only the clinical EC 3 isolate (colistin MIC ≥4 mg/L) contained the *mcr-1* gene, while all the other isolates were negative for the *mcr-1*, *mcr-2, mcr-3* and *mcr-4* genes.

**Table 1:** Colistin MICs and antimicrobial activity of biosurfactant extract ST34 (10.00 mg/mL) against a panel (*n* = 16) of MDR and XDR bacterial isolates

Organism	Strain Code	Source	Colistin MIC (mg/L)	Antimicrobial inhibition zone diameter (mm) ± SD
				Surfactin Extract
*A. baumannii*	AB 1	ATCC	2 (S)	13.3 ± 1.2
	AB 2	Clinical	2 (S)	14.7 ± 0.6
	AB 4	Clinical	> 4^a^ (R)	17.3 ± 0.6
	AB 6	Environmental	2 (S)	11.7 ± 1.2
*E. coli*	EC 1	ATCC	4 (R)	12.7 ± 0.6
	EC 2	Clinical	2 (S)	15.7 ± 0.6
	EC 3	Clinical	> 4^a^ (R)	15.7 ± 1.6
	EC 4	Environmental	4 (R)	14 ± 0
	EC 5	Environmental	4 (R)	17.7 ± 0.6
*K. pneumoniae*	KP 1	ATCC	4 (R)	14.3 ± 0.6
	KP 4	Clinical	4 (R)	13.3 ± 0.6
	KP 5	Environmental	> 4^a^ (R)	13 ± 1
*P. aeruginosa*	PA 1	ATCC	4 (R)	13 ± 0
	PA 2	Clinical	8 (R)	13 ± 0
	PA 3	Clinical	4 (R)	13.3 ± 0.6
	PA 4	Environmental	4 (R)	12.7 ± 0.6

^a^Corresponds to a colistin concentration exceeding 4 mg/L; S susceptible; R resistant

Table 1 Colistin MICs and antimicrobial activity of biosurfactant extract ST34 (10.00 mg/mL) against a panel (*n* = 16) of MDR and XDR bacterial isolates.

### Characterisation of Surfactin produced by the *B. amyloliquefaciens* ST34 strain using UPLC-ESI-MS

Ndlovu et al. [27] isolated the *B. amyloliquefaciens* ST34 strain from a WWTP in Stellenbosch. The spectra of the ST34 extract was thus compared to the surfactin standard (Sigma-Aldrich) and literature [27, 35]. The ion spectra in positive (ESI-MS) mode showed that the biosurfactant produced by ST34 displayed a profile similar to that of the surfactin standard (Additional file 1: Figure S2). The ESI-MS spectrum of the ST34 extract and the surfactin standard revealed five groups of analogue molecules (Additional file 1: Figure S2).

Table 2 Summary of the detected surfactin lipopeptides extracted from MSM cultured *B. amyloliquefaciens* ST34, as detected using UPLC-ESI-MS.

**Table 2:** Summary of the detected surfactin lipopeptides extracted from MSM cultured *B. amyloliquefaciens* ST34, as detected using UPLC-ESI-MS

Surfactin group	UPLC R_t_ (minutes)a	Proposed peptide sequences in surfactin group	Mono-isotopic [M] Exp/theor *M*_*r*_	Protonated Specie [M + H]^+^ Exp/theor *m/z*	Sodiated Specie [M + Na]^+^ Exp/theor *m/z*
Surfactin 1 (Srf1)	10.6; 11.2	cyclo[(**C**_**13**_**H**_**24**_**O**_**2**_)-L-Glu-**L-Leu**-D-Leu-L-Val-L-Asp-L-Leu-**L-Val**] cyclo[(**C**_**13**_**H**_**24**_**O**_**2**_)-L-Glu-**L-Ile**-D-Leu-L-Val-L-Asp-L-Leu-**L-Val**]	993.6376 993.6403	994.6472 994.6481	1016.6265 1016.6190
Surfactin 2 (Srf2)	11.0; 11.2; 11.9	cyclo[(**C**_**14**_**H**_**26**_**O**_**2**_)-L-Glu-**L-Leu**-D-Leu-L-Val-L-Asp-L-Leu-**L-Val**] cyclo[(**C**_**14**_**H**_**26**_**O**_**2**_)-L-Glu-**L-Ile**-D-Leu-L-Val-L-Asp-L-Leu-**L-Val**]	1007.6521 1007.6552	1008.6531 1008.6592	1030.6392 1030.6414
		cyclo-[(**C**_**13**_**H**_**24**_**O**_**2**_)-L-Glu-**L-Leu**-D-Leu-L-Val-L-Asp-L-Leu-**L-Leu**] cyclo[(**C**_**13**_**H**_**24**_**O**_**2**_)-L-Glu-**L-Leu**-D-Leu-L-Val-L-Asp-L-Leu-**L-Ile**] ^a^cyclo-[(**C**_**13**_**H**_**24**_**O**_2_)-L-Glu-**L-Ile**-D-Leu-L-Val-L-Asp-L-Leu-**L-Leu**] ^a^cyclo-[(**C**_**13**_**H**_**24**_**O**_**2**_)-L-Glu-**L-Ile**-D-Leu-L-Val-L-Asp-L-Leu-**L-Ile**]			
Surfactin 3 (Srf3)	11.6; 11.7; 12.3	cyclo[(**C**_**15**_**H**_**28**_**O**_**2**_)-L-Glu-**L-Leu**-D-Leu-L-Val-L-Asp-L-Leu-**L-Val**] cyclo[(**C**_**15**_**H**_**28**_**O**_**2**_)-L-Glu-**L-Ile**-D-Leu-L-Val-L-Asp-L-Leu-**L-Val**]	1021.6693 1021.6715	1022.6774 1022.6769	1044.6517 1044.6584
		cyclo[(**C**_**14**_**H**_**26**_**O**_**2**_)-L-Glu-**L-Leu**-D-Leu-L-Val-L-Asp-L-Leu-**L-Leu**] cyclo[(**C**_**14**_**H**_**26**_**O**_**2**_)-L-Glu-**L-Leu**-D-Leu-L-Val-L-Asp-L-Leu-**L-Ile**] ^a^cyclo-[(**C**_**14**_**H**_**26**_**O**_**2**_)-L-Glu-**L-Ile**-D-Leu-L-Val-L-Asp-L-Leu-**L-Leu**] ^a^cyclo-[(**C**_**14**_**H**_**26**_**O**_**2**_)-L-Glu-**L-Ile**-D-Leu-L-Val-L-Asp-L-Leu-**L-Ile**]			
Surfactin 4 (Srf4)	12.1; 12.2	cyclo[(**C**_**15**_**H**_**28**_**O**_**2**_)-L-Glu-**L-Leu**-D-Leu-L-Val-L-Asp-L-Leu-**L-Leu**] cyclo[(**C**_**15**_**H**_**28**_**O**_**2**_)-L-Glu-**L-Leu**-D-Leu-L-Val-L-Asp-L-Leu-**L-Ile**] ^a^cyclo[(**C**_**15**_**H**_**28**_**O**_**2**_)-L-Glu-**L-Ile**-D-Leu-L-Val-L-Asp-L-Leu-**L-Leu**] cyclo[(**C**_**15**_**H**_**28**_**O**_**2**_)-L-Glu-**L-Ile**-D-Leu-L-Val-L-Asp-L-Leu-**L-Ile**]	1035.6819 1035.6881	1036.6902 1036.6909	1058.6718 1058.6662
Surfactin 5 (Srf5)	12.6; 12.7	cyclo[(**C**_**16**_**H**_**30**_**O**_**2**_)-L-Glu-**L-Leu**-D-Leu-L-Val-L-Asp-L-Leu-**L-Leu**] ^a^cyclo[(**C**_**16**_**H**_**30**_**O**_**2**_)-L-Glu-**L-Leu**-D-Leu-L-Val-L-Asp-L-Leu-**L-Ile**] ^a^cyclo[(**C**_**16**_**H**_**30**_**O**_**2**_)-L-Glu-**L-Ile**-D-Leu-L-Val-L-Asp-L-Leu-**L-Leu**] ^a^cyclo[(**C**_**16**_**H**_**30**_**O**_**2**_)-L-Glu-**L-Ile**-D-Leu-L-Val-L-Asp-L-Leu-**L-Ile**]	1049.6992 1049.7032	1050.7036 1050.7066	1072.6941 1072.6886

R_t_ retention time, Theor theoretical *M*_*r*_, Exp Experimental *M*_*r*_, ^a^ UPLC Retention time of main peaks corresponding to the group’s *m/z* values

The positive mode spectra of the ST34 extract (Table 2) and the surfactin standard (results not shown) displayed five main groups of molecular ion *m/z* 994.65, 1008.66, 1022.68, 1036.69, and 1050.71 corresponding to the singly protonated charged species [M + H]^+^. Their corresponding sodium adducts [M + Na]^+^ were detected at *m/z* 1016.62, 1030.64, 1044.66, 1058.68 and 1072.69 (Table 2; Additional file 1: Figure S2). The surfactin species with longer fatty acyl chains were expected to elute later with a higher retention time (R_t_) [27]. This was observed in the UPLC-MS profile of the ST34 extracts with the surfactin groups eluting as follows; surfactin group 1 (Srf1) (R_t_ 10.6; 11.2 min), Srf2 (R_t_ 11.0; 11.2; 11.9 min), Srf3 (R_t_ 11.6; 11.7; 12.3 min), Srf4 (R_t_ 12.1; 12.2 min) and Srf5 (R_t_ 12.6; 12.7 min) (Additional file 1: Figure S2). The surfactin groups in the ST34 extract corresponding to the C_13_, C_14_, C_15_ and C_16_ (Srf1–5) analogues in the surfactin standard were also observed to have more than one retention time even though they displayed identical *m/z* and *M*_*r*_ values. The Srf3 and Srf4 analogues were present at the highest percentage of 42.1 and 34.9%, respectively, while the Srf1, Srf2 and Srf5 analogues contributed 5.6, 12.8 and 4.6% to the ST34 crude extract, respectively.

### Antimicrobial activity of Surfactin: agar disc susceptibility assay

The 16 isolates analysed for colistin resistance were also used as test strains for the assessment of the antimicrobial activity of the crude extract obtained from *B. amyloliquefaciens* ST34. Overall, antibacterial activity was observed against all test organisms utilised in this study (Table 1), with varying diameters for the zones of inhibition recorded.

For the *A. baumannii* isolates, the ST34 extract displayed the lowest activity against the environmental AB 6 isolate with a zone of inhibition of 11.7 ± 1.2 mm recorded, while the highest zone of inhibition was obtained against the XDR and colistin resistant clinical isolate AB 4 at 17.3 ± 0.6 mm (Table 1). An average zone of inhibition of 14.3 ± 2.3 mm was observed for the *A. baumannii* isolates. For *E. coli*, the highest zone of inhibition (17.7 ± 0.6 mm) was obtained for the environmental colistin resistant EC 5 isolate, while the lowest zone of inhibition was observed for the reference EC 1 isolate at 12.7 ± 0.6 mm (Table 1). Overall the *E. coli* isolates displayed an average zone of inhibition of 15.1 ± 1.8 mm. In contrast, the reference KP 1 isolate displayed the highest zone of inhibition at 14.3 ± 0.6 mm, while the environmental colistin resistant KP 5 isolate displayed the lowest zone of inhibition at 13 ± 1 mm (Table 1). The *K. pneumoniae* isolates displayed an average zone of inhibition of 13.6 ± 0.9 mm. All *P. aeruginosa* isolates displayed a similar sensitivity profile against the ST34 extract, with the clinical PA 3 isolate displaying the largest zone of inhibition at 13.3 ± 0.6 and the environmental PA 4 isolate displaying the smallest zone of inhibition at 12.7 ± 0.6 mm (Table 1). An average zone of inhibition of 13 ± 0.4 mm was observed for the *P. aeruginosa* isolates. In comparison to the ST34 extract, the commercial surfactin displayed comparable or lower activity against the reference *E. coli* EC 1, *K. pneumoniae* KP 1 and *P. aeruginosa* PA 1 strains, with no activity recorded against the *A. baumannii* AB 1 strain. No antimicrobial effect was observed for the negative control.

## Discussion

The efficacy of antibiotics is threatened by the rapid emergence of clinical and environmental bacterial strains exhibiting MDR and XDR profiles. The primary aim of the current study was thus to classify the antimicrobial resistance profiles of clinical, environmental and control strains of the WHO “Priority 1: Critical group” organisms, *A. baumannii*, *E. coli*, *K. pneumoniae* and *P. aeruginosa.* Subsequently, the susceptibility of the strains to various antibiotics, the last-resort antimicrobial, colistin and an alternative antimicrobial compound, surfactin, produced by a *B. amyloliquefaciens* ST34 strain, was determined.

An environmental *A. baumannii* strain was, however, not available to include in the analysis and was isolated from a stream located in a local informal settlement using growth media specific for *Campylobacter* species [32]. It is hypothesised that the isolation of *A. baumannii* could be attributed to the presence of antimicrobials in both the Modified Preston *Campylobacter* (polymyxin B, rifampicin, trimethoprim, and amphotericin B) and Modified Karmali (cefoperazone, vancomycin and amphotericin B) Selective Supplements. While the rifampicin, trimethoprim and polymyxin B are effective in inhibiting the growth of gram-negative bacteria, it should be noted that *A. baumannii* is known to exhibit rifampicin resistance [36, 37], trimethoprim resistance [37, 38] and remains viable at a polymyxin B concentration of 2 mg/L according to the CLSI [34] breakpoints. While this bacterium is classified as a strict aerobe and is predominantly associated with clinical settings [39], Fernando et al. [32] also recommended that, for the isolation of *A. baumannii* from environmental samples, cultures should be incubated under microaerophilic conditions. Culturing conditions employed by Fernando et al. [32] were thus successfully employed in the current study for the isolation of *A. baumannii* from environmental samples, contributing to the evidence of the existence of this pathogen in extra-hospital reservoirs. Antimicrobial susceptibility analysis was subsequently conducted to explore the resistance profiles of clinical, environmental and control strains of *A. baumannii*, *P. aeruginosa*, *E. coli* and *K. pneumoniae*.

Multidrug-resistant (MDR) strains of *A. baumannii* are resistant to three classes of antibiotics namely, β-lactam antibiotics (all penicillins and cephalosporins), fluoroquinolones and aminoglycosides, while extreme drug resistant (XDR) strains are resistant to the previously mentioned three classes of antibiotics as well as to carbapenems [40]. Results from the study then indicated that while the reference isolate AB 1 and the environmental isolate AB 6, displayed variable resistance, susceptibility and intermediate susceptibility profiles, the four clinical *A. baumannii* isolates (AB 2 – AB 5) were resistant > 90% of the antibiotics analysed (Additional file 1: Table S2) and were subsequently classified as XDR. A study by Chen et al. [41] similarly indicated that clinical *A. baumannii* isolates exhibit resistance to specifically β-lactams, fluoroquinolones, aminoglycosides and carbapenems. While further research is required, it is hypothesised that the XDR profile displayed by the clinical *A. baumannii* isolates can be attributed to the presence of multiple resistance mechanisms including β-lactamases [e.g. OXA-51-like enzymes OXA-51-like enzymes and a non-inducible chromosomal AmpC cephalosporinase (ADC1–7)] and aminoglycoside-modifying enzymes [e.g. acetyltransferase (AATs), nucleotidyltransferases (ANTs) and phosphotransferases (APHs)], membrane permeability alterations (e.g. loss of OMPs) or the overexpression or activation of multidrug efflux systems (e.g. AdeABC and AdeIJK) [2]. Moreover, antibiotic resistance in *A. baumannii* has been attributed to the alteration of the penicillin-binding proteins, DNA gyrase and topoisomerase IV mutations, tetracycline ribosomal protection protein and the involvement of dihydrofolate reductase in trimethoprim resistance [2, 4].

While based on the EUCAST [33] and CLSI [34] classification guidelines, the reference (EC 1), environmental (EC 4 to EC 7) and one clinical *E. coli* isolate (EC 3) were mostly susceptible to the antibiotics analysed (Fig. 1), the clinical *E. coli* isolate EC 2 exhibited resistance to β-lactams, fluoroquinolones and aminoglycosides and was characterised as MDR. The results obtained in the current study for EC 2 were also comparable to the study conducted by Yassin et al. [42], where clinical *E. coli* isolates were characterised as aztreonam, ceftazidime, cefotaxime and ciprofloxacin resistant. Moreover, while the clinical isolate EC 3 was resistant to ciprofloxacin [33], aztreonam and cefotaxime and both clinical isolates were resistant to tetracycline [34], it is hypothesised that the multidrug resistance displayed by the EC 2 isolate could also possibly be attributed to the presence of β-lactamases, aminoglycoside-modifying-enzymes and multidrug efflux pumps [43–45]. The β-lactamases and extended spectrum beta-lactamases (ESBLs) are primarily responsible for the hydrolysis of expanded spectrum cephalosporins (e.g. cefotaxime, cefepime and ceftazidime). Additionally, the AcrAB-TolC efflux pump, present in specifically MDR *E. coli* strains, can effectively remove fluoroquinolones and β-lactams from the cell [46].

Based on the antimicrobial susceptibility analysis obtained (Fig. 1), the reference KP 1 and clinical KP 2 and KP 3 isolates were also classified as MDR as they exhibited resistance to the three antibiotic classes β-lactams, fluoroquinolones and aminoglycosides. However, the clinical KP 4 isolate was classified as XDR as it exhibited resistance to the three antibiotic classes including carbapenems [imipenem (IMP)]. These results were comparable to a study conducted by Vasaikar et al. [46], where they characterised clinical *K. pneumoniae* isolates exhibiting cephalosporin and aminoglycoside resistance, amongst other antibiotics. Similar to *A. baumannii*, the resistance profile displayed by the MDR and XDR *K. pneumoniae* isolates may be attributed to the presence of multiple resistance mechanisms including β-lactamases [e.g. metallo-β-lactamases and expanded-spectrum oxacillinases (OXA-48)], aminoglycoside-modifying enzymes (e.g. AATs, ANTs and APHs) and the overexpression or activation of multidrug efflux systems (e.g. AcrAB multidrug efflux system) [2, 16, 47].

Results obtained also indicated that based on the EUCAST [33] breakpoints, the *P. aeruginosa* clinical isolate PA 3 should be classified as XDR as it exhibited resistance to β-lactams, fluoroquinolones, aminoglycosides and carbapenems. Penicillins (piperacillin-tazobactam or ticarcillin-clavulanate), cephalosporins (ceftazidime, cefepime or cefoperazone), monobactams (aztreonam), fluoroquinolones (ciprofloxacin or levofloxacin), carbapenems (imipenem, doripenem or meropenem), aminoglycosides (gentamicin, tobramycin or amikacin) and polymyxins (polymyxin B or colistin) are commonly used in the treatment of *P. aeruginosa* associated infections [47]. *Pseudomonas aeruginosa* is however, inherently resistant to a multitude of antibiotic classes including carbapenems, β-lactams, fluoroquinolones, aminoglycosides, and polymyxins [48, 49]. The primary antibiotic resistance mechanisms in *P. aeruginosa* have been identified as; the presence of β-lactamase enzymes (e.g. metallo-β-lactamases, AmpC, PSE-1 and PSE-4), aminoglycoside-modifying enzymes (e.g. AATs, ANTs and APHs), efflux systems (e.g. MexCD-OprJ, MexXY-OprM and MexAB-OprM) and permeability alterations (e.g. OprD porin deletion) [9, 50, 51].

The MIC of 16 representative MDR and XDR isolates (Table 1), against the last resort antibiotic colistin, was subsequently determined. These isolates were also screened for the mobilised colistin resistance genes (*mcr-1* to *mcr-4*). While a colistin MIC of ≥4 mg/L was recorded for 12 of the 16 isolates, only one strain (EC 3) was found to harbour a colistin resistance gene. It was also interesting to note that 75% of the *A. baumannii* and 20% of the *E. coli* strains analysed were still susceptible to colistin, while 100% of the *K. pneumoniae* and *P. aeruginosa* strains were resistant. Research has, indicated that resistance to colistin can be attributed to one of three possible mechanisms [3, 52]. The first involves the recently discovered colistin resistance mechanism associated with the *mcr* genes; the second mechanism involves the modification of the lipid A moiety of the lipopolysaccharide (LPS) mediated by mutation and/or over-expression in the two-component system *pmrA/pmrB* in response to environmental stimuli; while the third mechanism involves the complete loss of the LPS resulting from either a mutation of insertional inactivation of the lipid A biosynthesis genes *lpxA, lpxC* or *lpxD*. It is thus hypothesised that one or more of these mechanisms could have been expressed in the strains exhibiting colistin resistance.

Overall, results in the current study indicated that increased antibiotic and colistin resistance was observed amongst the clinical isolates of the “WHO Priority 1: Critical group” organisms, followed by the environmental and reference strains. Although antibiotic resistance is complex and multifactorial in nature, the increased resistance rates observed amongst the clinical isolates, in comparison to the environmental and reference isolates, could be attributed to the level of exposure of the pathogenic population to antimicrobials [53, 54]. Subsequently, the multitude of antimicrobials used in the clinical setting could have served as selectors for resistance genes, which are disseminated via horizontal gene transfer (HGT) or more specifically transformation, transduction and conjugation [53–55]. Furthermore, the prevalence of nosocomial infections caused by antibiotic resistant gram-negative bacteria has been associated with treatment failure, increased length of stay, significantly higher total hospital cost and increased mortality [56–58]. Cosgrove et al. [59] investigated the increased cost associated with the development of resistance during treatment. The authors found that patients infected with *Enterobacter* species, which developed expanded-spectrum cephalosporin resistance during antimicrobial treatment, had an average hospital stay of 9 days resulting in a higher hospital cost compared to the control group. Additionally, a study conducted by Kollef et al. [56] investigated the relationship between nosocomial infections that were not being effectively treated (termed inadequate antimicrobial therapies) and the emergence of infections caused by antibiotic resistant bacteria. Numerous corrective practices such as limiting the use of broad-spectrum antibiotics, applying antibiotic practice guidelines, rapid microbiological diagnostic methods and consulting infectious disease specialists, were outlined to curtail the development of antibiotic resistance in clinically important pathogens [56].

However, while the implementation of these clinical practices in restricting the incidence of nosocomial infections caused by antibiotic resistant bacteria is crucial, measures to combat MDR and XDR bacteria are still required. As indicated, Ndlovu et al. [27] isolated the *B. amyloliquefaciens* ST34 strain from a WWTP in Stellenbosch. The UPLC-MS spectra of the ST34 extract confirmed that the biosurfactant produced displayed a profile similar to that of the surfactin standard (Additional file 1: Figure S2). The ESI-MS spectrum of the ST34 extract and the surfactin standard revealed five groups of analogue molecules (Additional file 1: Figure S2). As research has indicated that lipopeptides such as surfactin, exhibit broad-spectrum antimicrobial activity [28], the susceptibility of the MDR and XDR WHO “Priority 1: Critical group” organisms, *A. baumannii*, *P. aeruginosa*, *E. coli* and *K. pneumoniae* strains to the crude surfactin extract produced by ST34 was determined. Antibacterial activity was observed against all the MDR and XDR strains in this study (Table 1), with varying diameters for the zones of inhibition recorded. Similar results were observed in a study conducted by Ndlovu et al. [27], where 100% antimicrobial activity of the surfactin extract obtained from *B. amyloliquefaciens* ST34, against a panel of gram-negative organisms, including antibiotic-resistant strains, was observed. A surfactin produced by the *B. amyloliquefaciens* M1 strain also exhibited broad-spectrum antibacterial activity against pathogens of the *Vibrio* species with MDR profiles [60]. Moreover, a surfactin produced by a *B. subtilis* strain was successfully shown to kill larval and pupal stages of the mosquito species *Anopheles stephensi, Aedesaegypti and Culexquinquefasciatus*, highlighting the potential of this biosurfactant as a prophylactic agent in the prevention of malaria [61]. Furthermore, while Hoefler et al. [62] observed resistance of test strains against surfactin (during the co-culturing of *B. subtilis* and *Streptomyces* species Mg1), which was ultimately attributed to the ability of *Streptomyces* to synthesise a surfactin hydrolysing enzyme, it is crucial to note that to date, limited resistance against various lipopeptides has been observed [30, 63]. Thus, while various clinical, environmental and control WHO Critical Priority List Bacteria were classified as MDR and XDR, based on their antibiograms, and colistin resistant, these isolates were susceptible to the surfactin crude extract produced by the *B. amyloliquefaciens* ST34 strain. Future studies will aim at purifying the surfactin homologues, determining the antimicrobial activity of the purified surfactin and subsequently determining the MIC’s.

## Conclusion

Clinical isolates of *A. baumannii*, *P. aeruginosa*, *E. coli* and *K. pneumoniae* exhibited increased antibiotic resistance profiles, compared to environmental and reference strains. Moreover, XDR and MDR isolates of clinical origin exhibited higher colistin resistance compared to environmental and references isolates. It was hypothesised that these resistant isolates were pre-exposed to the antibiotic classes and colistin in the clinical environments and may have acquired resistance through horizontal gene transfer. Results also indicated that the WHO Critical Priority List of Antibiotic-Resistant Bacteria are susceptible to surfactin produced by the *B. amyloliquefaciens* ST34 strain. Based on results obtained in the current study and previous research, this lipopeptide may possibly be exploited as an alternative antimicrobial against MDR or XDR gram-negative bacteria classified as “Priority 1: Critical group” organisms. To broaden the analysis of the antimicrobial activity of surfactin, the susceptibility of the “Priority 2: High” and “Priority 3: Medium” groups listed in the WHO Report [1] should also be determined. Future research should, however, focus on determining the cytotoxicity of surfactin analogues to elucidate the use of this biosurfactant as a chemotherapeutic agent and as a topical agent in the treatment of cutaneous or subcutaneous infections and for the coating or pre-treatment of medical equipment and devices for the prevention of biofilm formation.

## Methods

### Bacterial isolates

The *A. baumannii* [AB 1; ATCC (American Type Culture Collection) 19,606], *P. aeruginosa* (PA 1; ATCC 27853), *K. pneumoniae* (KP 1: ATCC 10031) and *E. coli* (EC 1; ATCC 13706) reference strains used in the current study were available in the Khan Laboratory Culture Collection (Additional file 1: Table S1). Clinical isolates of the respective organisms were obtained from either the Department of Biomedical Sciences at the Cape Peninsula University of Technology (CPUT) or the Department of Pathology (Division of Medical Microbiology), Stellenbosch University. In addition, environmental isolates of *K. pneumoniae*, *P. aeruginosa* and *E. coli* were obtained from the Khan Laboratory Culture Collection (Additional file 1: Table S1). These isolates were obtained from various environmental sources such as surface water, rainwater and solar pasteurized rainwater.

Environmental isolates of *A. baumannii* were however, not available in the Khan Laboratory Culture Collection and various environmental samples were collected for the isolation of this organism. Wastewater samples (1 L) were collected in sterile Schott bottles from the influent of the Stellenbosch Wastewater Treatment Plant (WWTP, GPS co-ordinates − 33.943505, 18.824584) in Stellenbosch, Western Cape as described by Ndlovu et al. [64]. Additionally, a 1 L water sample was collected from the Plankenburg River (GPS co-ordinates − 33.927761, 18.850544) and a 1 L surface run-off water sample was collected from a stream in Enkanini informal settlement (GPS co-ordinates − 33.924120; 18.847618) in Stellenbosch. Furthermore, sediment and soil samples were collected from the river and surface run-off stream by removing approximately 25 cm^2^ of topsoil and placing the soil in sterile 50 mL falcon tubes. To select for and isolate *A. baumannii,* an enrichment step was used as outlined by Fernando et al. [32]. Following primary enrichment, cultures (100 μL) were spread plated onto *Campylobacter* Agar Base (CAB) containing a Modified Karmali Selective Supplement [cefoperazone (32 mg/L), vancomycin (20 mg/L) and amphotericin B (10 mg/L)] (Oxoid, USA). The spread plates were incubated at 37 °C for 24–48 h under microaerophilic conditions. After the initial isolation, morphologically distinct colonies were further purified by streaking onto CAB and Nutrient Agar (NA; Biolab, Merck) to obtain pure cultures for identification.

### Genomic DNA extraction and conventional polymerase chain reaction

Genomic DNA was extracted from the isolates obtained from the environmental samples as well as all the reference, clinical and environmental Khan Laboratory Culture Collection isolates (Additional file 1**:** Table S1) using the boiling method as outlined by Ndlovu et al. [65].

Genomic DNA from all the reference, clinical and environmental isolates was used in genus or species-specific conventional PCR assays, as a quality control step, to identify all the isolates (Table 3). The PCR mixture for the identification of *A. baumannii*, *E. coli*, *K. pneumoniae* and *P. aeruginosa* isolates consisted of a final volume of 25 μL and contained: 1X Green GoTaq® Flexi buffer (Promega, Madison, Wi, USA); 0.75 U GoTaq® G2 DNA polymerase (*A. baumannii*) (Promega) or 1.5 U GoTaq® G2 DNA polymerase (*E. coli*, *K. pneumoniae* and *P. aeruginosa*) (Promega); 0.1 mM of the deoxynucleoside triphosphate (dNTP) mix (*A. baumannii*, *K. pneumoniae* and *P. aeruginosa*) (Thermo Scientific, Hudson) or 0.2 mM dNTP mix (*E. coli*) (Thermo Scientific); 2 mM MgCl_2_ (*A. baumannii*, *K. pneumoniae* and *P. aeruginosa*) (Promega) or 1.5 mM MgCl_2_ (*E. coli*) (Promega); 0.8 μM of the respective forward and reverse primers (Table 3, *A. baumannii* and *K. pneumoniae*), 0.2 μM of the respective forward and reverse primers (Table 3, *E. coli*) or 0.5 μM of the respective forward and reverse primers (Table 3, *P. aeruginosa*); with the following template DNA volumes – 2 μL (*E. coli)*, 2.5 μL (*A. baumannii*) and 5 μL (*K. pneumoniae* and *P. aeruginosa*). Sterile distilled H_2_O was used as a negative control, while the genomic DNA extracted from the *A. baumannii* ATCC 19606, *E. coli* ATCC 13706, *K. pneumoniae* ATCC 10031 and *P. aeruginosa* ATCC 27853 reference strains served as the positive controls. Amplification was performed according to the amplification conditions outlined in Table 3, using a T100™ Thermal Cycler (Bio-Rad Laboratories, Hercules, CA, USA).

**Table 3:** Primer sequences and PCR cycling conditions for the conventional PCR assays and for the identification of the mobilised colistin resistance genes

Bacteria or genes	Primer Name	Primer sequence (5′ → 3′)	PCR cycling conditions	Gene (size/bp)	Reference
*A. baumannii*	A.b_hyp F A.b_hyp R	CGTCGGTCGGATCCGTGTAT AAGTAAAGTGGCAGGCGCTT	95 °C for 5 min; 30 cycles of 94 °C for 30 s, 55 °C for 30 s, 72 °C for 45 s, 72 °C for 5 min	545	[66]
*E. coli*	PhoF PhoR	GTGACAAAAGCCCGGACACCATAAATGCCT TACACTGTCATTACGTTGCGGATTTGGCGT	94 °C for 2 min; 35 cycles of 94 °C for 1 min, 55 °C for 1 min and 72 °C for 1 min; 72 °C for 5 min	903	[67]
*Klebsiella* spp.	gryA-F gyrA-C	CGCGTACTATACGCCATGAACGTA ACCGTTGATCACTTCGGTCAGG	95 °C for 3 min; 35 cycles of 94 °C for 1 min, 50 °C for 30 s and 72 °C for 30 s; 72 °C for 5 min	383	[68]
*Pseudomonas* spp.	PA-GS-F PA-GS-R	GACGGGTGAGTAATGCCTA CACTGGTGTTCCTTCCTATA	95 °C for 2 min; 25 cycles of 94 °C for 20 s, 54 °C for 20 s and 72 °C for 40 s; 72 °C for 5 min	618	[69]
*mcr-1*	mcr1_320bp_fw mcr1_320bp_rev	AGTCCGTTTGTTCTTGTGGC AGATCCTTGGTCTCGGCTTG	94 °C for 15 min, 25 cycles at 94 °C for 30 s, 58 °C for 90 s, 72 °C for 60 s, 72 °C for 10 min	320	[19]
*mcr-2*	mcr2_700bp_fw mcr2_700bp_rev	CAAGTGTGTTGGTCGCAGTT TCTAGCCCGACAAGCATACC		715	
*mcr-3*	mcr3_900bp_fw mcr3_900bp_rev	AAATAAAAATTGTTCCGCTTATG AATGGAGATCCCCGTTTTT		929	
*mcr-4*	mcr4_1100bp_fw mcr4_1100bp_rev	TCACTTTCATCACTGCGTTG TTGGTCCATGACTACCAATG		1116	

Following amplification, the PCR products were analysed by gel electrophoreses [80 Volts (V) for 60 min] using 0.8% agarose (SeaKem® LE Agarose; Lonza) containing 0.5 μg/mL ethidium bromide in a 1X Tris borate ethylenediaminetetraacetic acid (TBE) buffer. Once the DNA band size of the PCR products were confirmed, representative PCR products were purified and concentrated using the Wizard® SV Gel and PCR Clean-up System (Promega) according to the manufacturer’s instructions. The cleaned PCR products were sent to the Central Analytical Facility (CAF) at Stellenbosch University for sequencing using the BigDye Terminator Version 3.1 Sequencing Kit (Applied Biosystems®, Foster City, USA). The chromatograms of each sequence were examined using FinchTV version 1.4.0 software and sequence identification was completed using the National Center for Biotechnology Information (NCBI), Basic Local Alignment Search Tool (BLAST), available at http://blast.ncbi.nlm.nih.gov/Blast.cgi [70].

Table 3 Primer sequences and PCR cycling conditions for the conventional PCR assays and for the identification of the mobilised colistin resistance genes.

### Kirby-Bauer antibiotic assays

To determine the antibiotic resistance profiles of all the clinical, environmental and reference strains, the organisms were re-streaked from either glycerol stocks (40% glycerol stored at − 80 °C) or CAB plates onto NA and were incubated at 37 °C for 18–24 h. Subsequently, all the isolates were inoculated into 10 mL LB broth and were grown overnight at 37 °C. Following the overnight incubation, the optical density (OD) of the bacterial suspensions were measured using a T60 Visible Spectrophotometer (PG-Instruments Limited, Leicester, UK) at 625 nm. The OD_625_ of the suspensions was adjusted using LB broth to 0.08–0.1, which corresponded to approximately 1.5 × 10^8^ colony forming units (CFU)/mL (EUCAST) [33]. One hundred microlitres of each adjusted suspension was subsequently spread plated onto Mueller-Hinton agar (not cation-adjusted) (MHA, Biolab, Merck). Commercially-prepared, fixed concentration, antibiotic discs (Oxoid, USA) [e.g. amikacin (30 μg), ampicillin (10 μg), aztreonam (30 μg), cefepime (30 μg), cefotaxime (30 μg), ceftazidime (30 μg), ciprofloxacin (5 μg), gentamicin (10 μg), imipenem (10 μg), levofloxacin (5 μg), meropemen (10 μg), piperacillin-tazobactam (103.9 / 11.3 μg) and tetracycline (30 μg)] were placed onto the surface of the agar media [71].The respective bacterial isolates were subjected to various antibiotics as outlined in Additional file 1: Table S2, with zones of inhibition delineated by either the EUCAST [33] or CLSI [34] guidelines. All the antibiotic disc assays were performed in triplicate and the plates were incubated at 37 °C for 18–24 h. Following incubation, the diameter of the zone of inhibition around each antibiotic disc was measured to the closest millimetre. These measurements were compared to the clinical breakpoints as outlined in the EUCAST [33] and CLSI [34] standards to determine if the organisms were resistant, intermediately susceptible or susceptible to the tested antibiotics. Based on the resistance profiles, isolates were classified as multidrug resistant (MDR) or extensively resistant (XDR). Multidrug resistance (MDR) referred to resistance to three classes of antibiotics namely β-lactam antibiotics (all penicillins and cephalosporins), fluoroquinolones and aminoglycosides, whereas XDR referred to the resistance to the previously mentioned three classes of antibiotics as well as to carbapenems [40].

### Colistin minimum inhibitory concentration

Based on the results obtained for the antibiotic disc assays, isolates displaying antibiotic resistance were further screened for colistin resistance using the methodology described by the EUCAST [33] and CLSI [34]. Serial dilutions of the colistin sulphate stock solution (10.00 mg/mL) (VWR Life Sciences, Philadelphia, USA) were prepared in milliQ water corresponding to a concentration range of 0.06 mg/L to 4 mg/L for *A. baumannii*, *E. coli* and *K. pneumoniae* and 0.06 mg/L to 8 mg/L for *P. aeruginosa* [33]. Cell suspensions of the isolates were prepared and diluted in LB broth to obtain an OD_625_ of 0.08–0.1 [33]. One hundred microlitres of cation-adjusted Mueller–Hinton Broth 2 (CAMHB; Biolab, Merck) and 100 μL of each of the colistin dilutions were pipetted into the wells of a Nunclon™ Delta Surface 96-well tissue culture microtitre plate (Nunc™, Roskilde, Denmark). Following the addition of the CAMHB and colistin, 10 μL of the adjusted OD bacterial inoculums was added to the respective wells. In addition, a positive control (organism in CAMHB without colistin) and a sterility control (CAMHB) were included for each minimum inhibitory concentration (MIC) assay. *Escherichia coli* EC3 (colistin resistant) and *P. aeruginosa* ATCC 27853 (colistin sensitive) were included in all assays as quality controls. The 96-well plates were incubated at 35 ± 2 °C for 20–24 h. The susceptibility of the organisms to colistin was determined by measuring the optical density of the samples at a wavelength of 600 nm (OD_600_) before (t = 0) and after incubation (t = 24) using a PowerWave™ Microplate Spectrophotometer (BioTek Instruments, Vermont, USA). The percentage inhibition was calculated as outlined in Poimenidou et al. [72]. The MIC was determined as the minimum concentration of the antimicrobial solution at which no increase of OD (OD_600_ ~ 0 at t = 24) was observed. Subsequently, the MIC was compared to the EUCAST [33] and CLSI [34] breakpoints to determine the organism’s susceptibility or resistance to colistin.

### Multiplex PCR for Colistin resistance genes

All the bacterial isolates employed in the current study (Additional file 1: Table S1) were screened for the plasmid-mediated mobilised colistin resistance (*mcr-1* to *mcr-4*) genes as described by Rebelo et al. [19]. To confirm the presence of the *mcr-1*, *mcr-2*, *mcr-3* or *mcr-4* plasmid-mediated resistance genes, plasmid isolation was performed for each of the colistin resistant organisms using the PureYield™ Plasmid Miniprep System (Promega, South Africa), according to the manufacturer’s instructions. For the amplification of the *mcr-1* to *mcr-4* genes, each multiplex PCR mixture consisted of a final volume of 25 μL, containing 1X Green GoTaq® Flexi buffer, 2 mM MgCl_2_ (Promega), 0.1 mM of a dNTP mix (Thermo Scientific), 0.2 μM of the respective forward and reverse PCR primers (Table 3), 0.75 U GoTaq® Flexi DNA polymerase (Promega) and 2 μL plasmid DNA, per respective isolate. Amplification was performed using a T100™ Thermal Cycler (Bio-Rad Laboratories, USA) using the PCR amplification conditions outlined in Table 3. Plasmid DNA extracted from the *E. coli* Tygerberg Hospital isolate (EC 3; *mcr-1* positive) and the *P. aeruginosa* ATCC 27853 isolate (PA 1; *mcr-1* negative control) served as the positive and negative controls for the *mcr-1* gene. Sterile distilled H_2_O served as the PCR negative control. The obtained PCR products were visualised, purified, concentrated and sent to CAF for sequencing as previously described.

### Extraction, purification and characterisation of Surfactin produced by the *B. amyloliquefaciens* ST34 strain

The *B. amyloliquefaciens* ST34 strain (collection number SARCC 696 at the South African Rhizobium Culture Collection), isolated by Ndlovu et al. [64] from the Stellenbosch WWTP in the Western Cape, South Africa, was used to obtain the antimicrobial lipopeptide surfactin. The methods previously described by Ndlovu et al. [27, 64] were used for the production, extraction and characterisation of the crude extract obtained from *B. amyloliquefaciens* ST34. Briefly, *B. amyloliquefaciens* ST34 was streaked onto NA plates which were incubated at 37 °C for 18–24 h. Seed cultures were prepared in 5 mL sterile mineral salt medium (MSM; 0.1 KH_2_PO_4_, 0.1% K_2_HPO_4_, 0.02% MgSO_4_·7H_2_O, 0.002% CaCl_2_·2H_2_O, 0.005% FeCl_3_·6H_2_O and 0.2% NaNO_3_ and 3% glycerol, with the pH adjusted to 6.8) with a single colony from the NA cultures [27, 64]. The seed cultures were cultivated at 30 °C and 200 rpm for 18–24 h.

Following the preparation of the seed cultures, a 500 mL baffled flask containing 100 mL MSM was inoculated with a 2% cell suspension (OD_600_ = 0.7). Broth cultures were incubated on an orbital shaker (New Brunswick, NY, USA) at 120 rpm for 120 h at 30 °C. After 5 days, the cultures (100 mL) were centrifuged at 11305×*g* for 30 min at 4 °C to remove microbial cells. The supernatant was removed and subsequently acidified using hydrochloric acid (Merck, Darmstadt, Germany) to a pH of 7.5, whereafter it was stored at 4 °C to induce the precipitation of the biosurfactant. Using centrifugation at 11305×*g* for 30 min at 4 °C the precipitate was harvested and washed with 50 mL milliQ water, thereafter the sample pH was adjusted to 7.5. Insoluble fractions were lyophilised using a freeze dryer (Virtis Bechtop K, SP-Industries, NY, USA) and were further extracted using 70% acetonitrile (Romil, Waterbeach, England). This process was repeated thrice. Following lyophilisation, crude extracts were stored at − 20 °C until characterisation.

Mass spectrometry analysis was performed at the CAF of Stellenbosch University using a Waters Quadrupole Time of Flight Synapt G2 (Waters Corporation, Milford, USA) mass spectrometer. For UPLC-MS analysis, 3 μL of the commercial surfactin standard (Sigma-Aldrich) and the ST34 crude extract was injected and separated on a UPLC C18 reverse-phase analytical column (Acquity UPLC® HSS T3, 1.8 μm particle size, 2.1 × 150 mm, Waters corporation, Dublin, Ireland) at a flow rate of 0.300 mL/minute using 0.1% formic acid (A) to acetonitrile (B) gradient [60% (A) from 0 to 0.5 min for loading; a 40 to 95% gradient (B) from 0.5 to 11 min and then 95 to 40% (B) from 15 to 18 min] [65]. A capillary voltage of 3 kV, cone voltage of 15 V and a temperature of 120 °C was utilised during the UPLC-MS analysis of the analytes [27]. The data acquisition was conducted in positive mode for the MS-scanning of a second analyser through the *m/z* range of 200–3000 Da. Data were analysed using Masslynx software version 4.1 (Water Corporation, Milford, USA) and the UPLC-MS profiles obtained for the crude extract was compared to the surfactin (Sigma-Aldrich).

### Antimicrobial activity of Surfactin: agar disc susceptibility test

The susceptibility of the reference, clinical and environmental bacterial strains to the crude extract (10.00 mg/mL) obtained from the *B. amyloliquefaciens* ST34 strain was determined using the method outlined by Ndlovu et al. [27]. Briefly, the lyophilised ST34 crude extract was dissolved in 15% (v/v) methanol to obtain a concentration of 10.00 mg/mL. Antimicrobial discs (6 mm; Oxoid, USA) were placed on the MHA after the test isolates were spread plated onto the media and were impregnated with 50 μL of the surfactin crude extract (with approximately 9.98 μg surfactin in the crude extract). For each assay, a surfactin negative and positive control was included in the experiment for each of the test organisms. The negative control consisted of the test microorganism spread plated onto MHA (Merck) with 15% (v/v) methanol impregnated filter paper discs, while the positive control consisted of the reference test organisms [AB 1, EC 1, KP 1 and PA 1] spread plated onto MHA with commercial surfactin (1.00 mg/mL) (Sigma, USA) impregnated filter paper discs added on top of the media. All the agar plates were incubated for 24–48 h at 37 °C, whereafter the diameters of the zones of inhibition around the inoculated paper discs were measured and recorded.

## Supplementary information

**Additional file 1: Table S1**. Bacterial isolates utilised as test organisms in this study. **Table S2**. Antibiotics used in the disc diffusion assay against test organism. Antibiotics selected for the Kirby-Bauer antibiotic assays based on the availability of EUCAST [33] or CLSI [34] breakpoint data. **Figure S1.** Antibiotic susceptibility assays of four representative isolates, A) *A. baumannii* AB1 (MEM^1^, CTX^2^, IMP^3^ and CIP^4^), B) *E. coli* EC1 (CTX^1^, FEP^2^, LEV^3^ and IMP^4^), C) *K. pneumoniae* KP1 (LEV^1^, FEP^2^, CN^3^ and AK^4^) and D) *P. aeruginosa* PA1 (IMP^1^, AK^2^, LEV^3^ and MEM^4^). The antibiotic susceptibility assay of four represented isolates (*A. baumannii*, *E. coli*, *K. pneumoniae* and *P. aeruginosa)* exposed to various antibiotics indicated in brackets. **Figure S2.** The positive mean spectrum of the ESI-MS analysis generated with MaxEnt3 of the surfactin standard (A) and ST34 MSM extract (B) is shown. Masses are indicated as [M_r_ + H] = *m/z* of singly charged species. Refer to Table 2 for identities of Srf1 – Srf5 and the expected *m/z* and *M*_*r*_ values. The positive mean spectrum of the ESI-MS analysis generated with MaxEnt3 of the surfactin standard (A) and ST34 MSM extract (B) is shown. Masses are indicated as [M_r_ + H] = *m/z* of singly charged species.

## Data Availability

The datasets used and/or analysed during the current study are available from the corresponding author on reasonable request.

## Abbreviations

AB: *Acinetobacter baumannii*
ACC: Aminoglycoside acetyltransferase
AK: Amikacin
AMP: Ampicillin
AMU: Atomic mass units
ANT: Aminoglycoside nucleotidyltransferase
APH: Aminoglycoside phosphoryl transferase
ATCC: American Type Culture Collection
ATM: Aztreonam
BLAST: Basic Local Alignment Search Tool
CAB: *Campylobacter* Agar Base
CAF: Central Analytical Facility
CAMHB: Cation-adjusted Mueller Hinton Broth
CAZ: Ceftazidime
CFU: Colony forming units
CIP: Ciprofloxacin
CLSI: Clinical and Laboratory Standard Institute
CN: Gentamicin
CPUT: Cape Peninsula University of Technology
CTX: Cefotaxime
DNA: Deoxyribonucleic acid
dNTP: Deoxynucleoside triphosphate
EC: *Escherichia coli*
ESBL: Extended spectrum beta lactamases
ESI-MS: Electrospray ionisation mass spectrometry
EUCAST: European Committee on Antimicrobial Testing
FEP: Cefepime
HGT: Horizontal gene transfer
ICU: Intensive care unit
IMP: Imipenem
KP: *Klebsiella pneumoniae*
LB: Luria Bertani Broth
LEV: Levofloxacin
LPS: Lipopolysaccharides
*m/z*: Mass-to-charge ratio
*mcr*: Mobilised colistin resistant
MDR: Multidrug resistant
MEM: Meropenem
MHA: Mueller Hinton Agar
MHB: Mueller Hinton Broth
MIC: Minimum inhibitory concertation
*M*_*r*_: Molecular mass
MRSA: Methicillin resistance *S. aureus*
MSM: Mineral salt medium
NA: Nutrient Agar
NCBI: National Center for Biotechnology Information
OD: Optical density
OMP: Outer membrane protein PA *Pseudomonas aeruginosa*
PCR: Polymerase chain reaction
R_t_: Retention time
SARCC: South African Rhizobium Culture Collection
SD: Standard deviation
Srf: Surfactin group
TBE: Tris Borate Ethylenediaminetetraacetic acid
TE: Tetracycline
TZP: Piperacillin-tazobactam
UPLC: Ultra-performance liquid chromatography
UTI: Urinary tract infection
WHO: World Health Organisation
WWTP: Wastewater treatment plant
XDR: Extreme drug resistant
