# Supplementary material for: Exploring the antimicrobial resistance profiles of WHO critical priority list bacterial strains
Source: BMC Microbiol. 2019 Dec 23;19:303. doi: 10.1186/s12866-019-1687-0 (PMC6929480; doi:10.1186/s12866-019-1687-0)
Supplement: Supplementary file 1 — Additional file 1: Table S1. Bacterial isolates utilised as test organisms in this study. Table S2. Antibiotics used in the disc diffusion assay against test organism. Antibiotics selected for the Kirby-Bauer antibiotic assays based on the availability of EUCAST [33] or CLSI [34] breakpoint data. Figure S1. Antibiotic susceptibility assays of four representative isolates, A) A. baumannii AB1 (MEM1, CTX2, IMP3 and CIP4), B) E. coli EC1 (CTX1, FEP2, LEV3 and IMP4), C) K. pneumoniae KP1 (LEV1, FEP2, CN3 and AK4) and D) P. aeruginosa PA1 (IMP1, AK2, LEV3 and MEM4). The antibiotic susceptibility assay of four represented isolates (A. baumannii, E. coli, K. pneumoniae and P. aeruginosa) exposed to various antibiotics indicated in brackets. Figure S2. The positive mean spectrum of the ESI-MS analysis generated with MaxEnt3 of the surfactin standard (A) and ST34 MSM extract (B) is shown. Masses are indicated as [Mr + H] = m/z of singly charged species. Refer to Table 2 for identities of Srf1 – Srf5 and the expected m/z and Mr values. The positive mean spectrum of the ESI-MS analysis generated with MaxEnt3 of the surfactin standard (A) and ST34 MSM extract (B) is shown. Masses are indicated as [Mr + H] = m/z of singly charged species. [file 12866_2019_1687_MOESM1_ESM.docx]

# **Additional file 1**

**Table S1.** Bacterial isolates utilised as test organisms in this study

| **Organism** | **Strain Code** | **Source** | **GenBank Accession number** | **% ID** |
| --- | --- | --- | --- | --- |
| *Acinetobacter baumannii* | AB 1 | ATCC | CP030083.1 | 99 |
| *Acinetobacter baumannii* | AB 2 | Clinical | CP030106.1 | 99 |
| *Acinetobacter baumannii* | AB 3 | Clinical | CP030106.1 | 97 |
| *Acinetobacter baumannii* | AB 4 | Clinical | CP030106.1 | 100 |
| *Acinetobacter baumannii* | AB 5 | Clinical | CP030106.1 | 100 |
| *Acinetobacter baumannii* | AB 6 | Environmental | CP030106.1 | 100 |
| *Escherichia coli* | EC 1 | ATCC | CP029180.1 | 99 |
| *Escherichia coli* | EC 2 | Clinical | CP028483.1 | 99 |
| *Escherichia coli* | EC 3 | Clinical | CP027255.1 | 99 |
| *Escherichia coli* | EC 4 | Environmental | CP029122.1 | 99 |
| *Escherichia coli* | EC 5 | Environmental | CP024243.1 | 99 |
| *Escherichia coli* | EC 6 | Environmental | CP028122.1 | 99 |
| *Escherichia coli* | EC 7 | Environmental | CP029574.1 | 99 |
| *Klebsiella pneumoniae* | KP 1 | ATCC | HM452918.1 | 98 |
| *Klebsiella pneumoniae* | KP 2 | Clinical | CP029388.1 | 99 |
| *Klebsiella pneumoniae* | KP 3 | Clinical | CP023441.1 | 99 |
| *Klebsiella pneumoniae* | KP 4 | Clinical | CP029388.1 | 99 |
| *Klebsiella pneumoniae* | KP 5 | Environmental | MF741938.1 | 99 |
| *Klebsiella pneumoniae* | KP 6 | Environmental | HM452918.1 | 98 |
| *Klebsiella pneumoniae* | KP 7 | Environmental | MF741930.1 | 99 |
| *Pseudomonas aeruginosa* | PA 1 | ATCC | MH817477.1 | 97 |
| *Pseudomonas aeruginosa* | PA 2 | Clinical | MH817477.1 | 99 |
| *Pseudomonas aeruginosa* | PA 3 | Clinical | KY582738.1 | 97 |
| *Pseudomonas aeruginosa* | PA 4 | Environmental | MH828325.1 | 100 |
| *Pseudomonas aeruginosa* | PA 5 | Environmental | MH817477.1 | 99 |

ATCC American Type Culture Collection, CPUT Cape Peninsula University of Technology, % ID Percentage sequence similarity between the query sequence and the reference sequence on the National Center for Biotechnology Information (NCBI) database

**Table S2.** Antibiotics used in the disc diffusion assay against test organism

| ***A. baumannii*** | ***E. coli*** | ***K. pneumoniae*** | ***P. aeruginosa*** |
| --- | --- | --- | --- |
| Amikacin | Amikacin | Amikacin | Amikacin |
| NT* | Ampicillin | Ampicillin | NT* |
| NT* | Aztreonam | Aztreonam | Aztreonam |
| Cefepime | Cefepime | Cefepime | Cefepime |
| Cefotaxime | Cefotaxime | Cefotaxime | NT* |
| Ceftazidime | Ceftazidime | Ceftazidime | Ceftazidime |
| Ciprofloxacin | Ciprofloxacin | Ciprofloxacin | Ciprofloxacin |
| Gentamicin | Gentamicin | Gentamicin | Gentamicin |
| Imipenem | Imipenem | Imipenem | Imipenem |
| Levofloxacin | Levofloxacin | Levofloxacin | Levofloxacin |
| Meropenem | Meropenem | Meropenem | Meropenem |
| Piperacillin-tazobactam | Piperacillin-tazobactam | Piperacillin-tazobactam | Piperacillin-tazobactam |
| Tetracycline | Tetracycline | Tetracycline | NT* |

NT* Antibiotic not tested


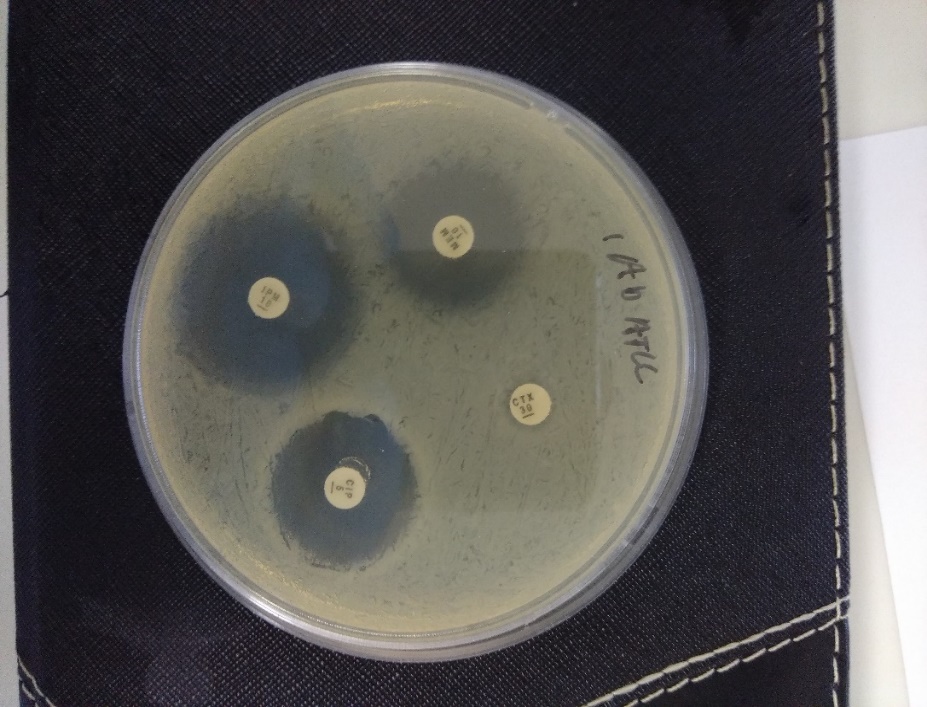

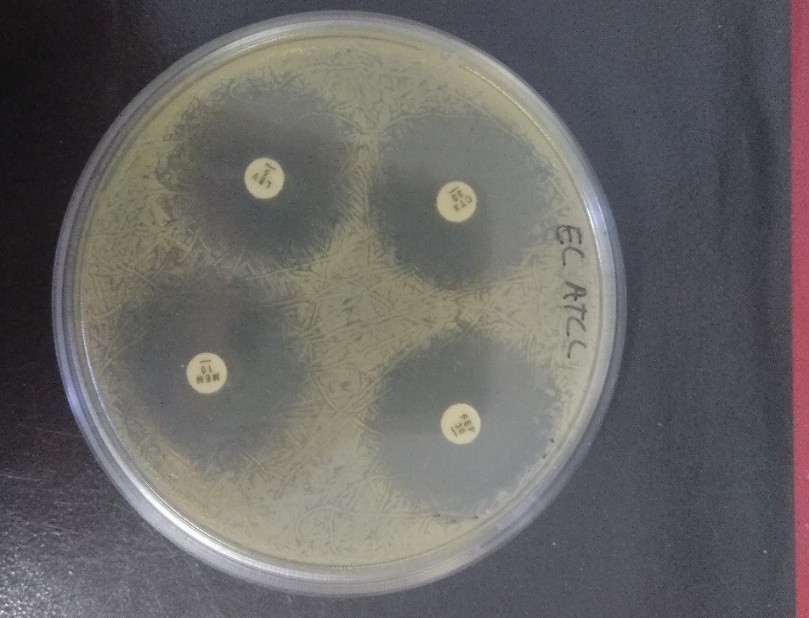

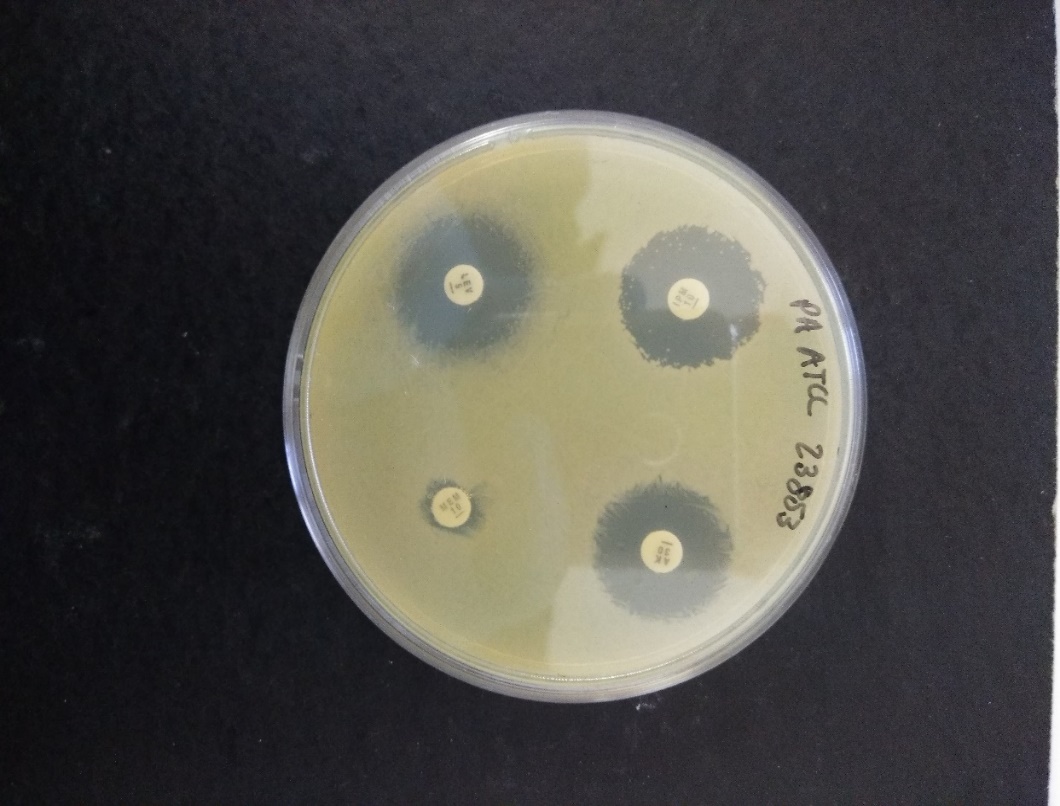

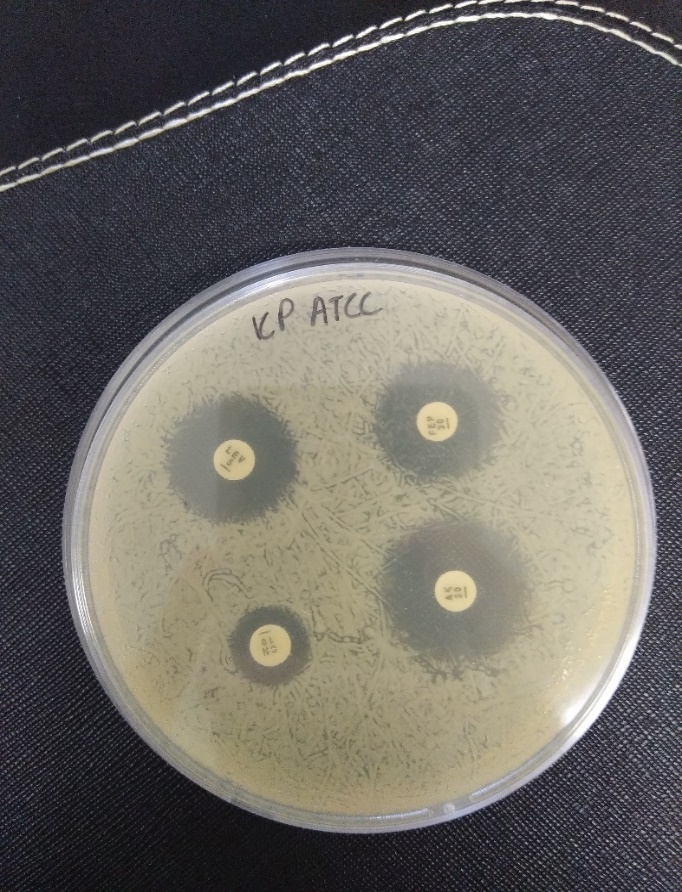


A

B

C

D

1

2

3

4

1

2

3

4

1

2

3

4

1

2

3

4

**Fig. S1**. Antibiotic susceptibility assays of four representative isolates, A) *A. baumannii* AB1 (MEM^1^, CTX^2^, IMP^3^ and CIP^4^), B) *E. coli* EC1 (CTX^1^, FEP^2^, LEV^3^ and IMP^4^), C) *K. pneumonia*e KP1 (LEV^1^, FEP^2^, CN^3^ and AK^4^) and D) *P. aeruginosa* PA1 (IMP^1^, AK^2^, LEV^3^ and MEM^4^)

A

mass

950

960

970

980

990

1000

1010

1020

1030

1040

1050

1060

1070

1080

1090

1100

%

0

100

Srf1+H

1022.6757

994.6420

980.6349

955.7398

962.7205

1009.6641

1000.2821

1016.6269

1036.6934

1030.6394

1050.7023

1044.6578

1058.6744

1060.6473

1072.6906

1088.6702

Srf3+H

Srf2+H

Srf4+H

Srf5+H

Srf2+Na

Srf3

+Na

Srf1+Na

Srf5+Na

1008.6559

Srf4+Na

mass

950

960

970

980

990

1000

1010

1020

1030

1040

1050

1060

1070

1080

1090

1100

%

0

100

Srf1

1022.6780

998.2856

994.6492

983.6477

950.6865

965.6909

1008.6586

1019.2122

1036.6945

1030.6440

1044.6610

1058.6766

1050.7074

1060.6483

1074.6517

1072.6952

1093.8265

B

Srf2

Srf3

Srf4

Srf5

Srf2+Na

Srf3+Na

Srf5+Na

Srf4+Na

**Fig. S2.** The positive mean spectrum of the ESI-MS analysis generated with MaxEnt3 of the surfactin standard (A) and ST34 MSM extract (B) is shown. Masses are indicated as [M_r_ + H] = m/z of singly charged species. Refer to **Table 3** for identities of Srf1 – Srf5 and the expected *m/z* an *M_r_* values
